# The Frequency of Anemia and Underlying Factors among Iranian Pregnant Women from Provinces with Different Maternal Mortality Rate

**Published:** 2019-02

**Authors:** Mohammad Esmaeil MOTLAGH, Seiyed Davoud NASROLLAHPOUR SHIRVANI, Farahnaz TORKESTANI, Zahra HASSANZADEH-ROSTAMI, Seyed-Mozaffar RABIEE, Hassan ASHRAFIAN AMIRI, Laleh RADPOOYAN

**Affiliations:** 1. Department of Pediatrics, School of Medicine, Ahvaz Jundishapur University of Medical Sciences, Ahvaz, Iran; 2. Social Determinants of Health Research Center, Health Research Institute, Babol University of Medical Sciences, Babol, Iran; 3. Department of Obstetrics and Gynecology, School of Medicine, Shahed University, Tehran, Iran; 4. Department of Community Nutrition, School of Nutrition and Food Sciences, Shiraz University of Medical Sciences, Shiraz, Iran; 5. Department of Anesthesiology and Intensive Care, Babol University of Medical Sciences, Babol, Iran; 6. Internal Medicine, Babol University of Medical Sciences, Babol, Iran; 7. Department of Health, Ministry of Health, Tehran, Iran

**Keywords:** Anemia, Hemoglobin, Pregnancy, Risk factor, Delivery of health care

## Abstract

**Background:**

Anemia is a common nutritional disorder that is more prevalent in pregnant women than other population groups. This study aimed to assess the frequency of anemia and its association with health care determinants among Iranian pregnant women from provinces with different Maternal Mortality Rate (MMR).

**Methods:**

This cross-sectional survey was carried out on 2737 pregnant women referred to public health centers in Iran, 2015. The participants were randomly selected by multistage sampling from six provinces with low, moderate or high MMR. The level of hemoglobin lower than 11 g/dl were defined as anemia in first and third trimester of pregnancy.

**Results:**

The rate of anemia in first and third trimester were respectively 8.2 and 26.7%. The most determinants of anemia among women in both first and third trimester of pregnancy were geographical classes with high MMR, no care before pregnancy, and type of house. Moreover, lower number of previous pregnancies (OR, 0.48; 95% CI, 0.27 to 0.85) and adequate care during pregnancy (OR, 0.66; 95% CI, 0.47 to 0.92) were protected women from anemia and high number of children (OR, 2.07; 95% CI, 1.13 to 3.80) enhanced risk of anemia in first trimester of pregnancy. Moreover, higher body mass index had lower odds of anemia in third trimester.

**Conclusion:**

The rate of anemia is differed in various parts of Iran, and this disorder gets worse in third trimester of pregnancy than first. Strengthening health care programs may be a useful strategies to prevent and control anemia.

## Introduction

Anemia is a public health problem estimated more than 1.62 billion people are facing this disorder (1). Although all age groups are predisposed to anemia, pregnant women have been identified as a vulnerable group prone to anemia and its complications (2). During pregnancy, the anemia intensifies due to enhancement of plasma volume and decrement of hemoglobin concentration (3). In addition to maternal health, fetus is also affected in pregnant anemic women. The progression of anemia, especially in last months of pregnancy, result fetal complications such as preterm delivery, low birth weight and stillbirth (4, 5).

Anemic pregnant women are almost four times less tolerate to bleeding, that may higher risk of mortality during childbirth. Thus, high percentage of mortality during pregnancy and delivery could be due to anemia, compared to other complications (6). The rate of anemia in developing countries is higher than developed ones, due to different demographic characteristics, lifestyle pattern and diet diversity. WHO reported more than half of women in developing countries are anemic which is higher in pregnant women (7, 8).

Several determinants of anemia in pregnant women including; anthropometric indices, gestational age, mode of previous childbirth, history of anemia in last pregnancy, iron supplementation, number of previous pregnancies, history of abortion, history of hemorrhage in previous pregnancy, type of pregnancy (wanted or unwanted), and etc. However, the impact of these factors reported differently in various regions of the world (9). Therefore, the prevalence of anemia differs according to regions, cultures, diet diversity and demographic characteristics.

On the other hand, educational interventions could effectively prevent and control anemia by considering underlying factors specified to each population and regions.

Given the lack of update and comprehensive data on rate of anemia in Iranian pregnant women, this study aimed to estimate the frequency of anemia in pregnant women and determine its underlying factors in six provinces of Iran with different Maternal Mortality Rate (MMR).

## Methods

This nationwide cross-sectional survey was conducted in autumn, 2015 on Iranian pregnant women. We used multistage random sampling method. At first level, we categorized provinces in 3 levels; low MMR (lower than 15 maternal deaths in 100000 childbirth), moderate MMR (between 15–25 maternal deaths in 100000 childbirth), and high MMR (higher than 25 maternal deaths in 100000 childbirth), according to a national study during 2007–2012 (10).

Then six provinces were randomly selected, with considering 2 provinces in each category. The sample size in each province was estimated 400, based on predicted prevalence of anemia to be around 0.5% and considering 95% confidence interval and ±5% error. The random sampling was conducted in 24 health centers in urban and rural areas of selected provinces.

We obtained a verbal consent from all participants after explaining the goal of study. This study was approved by Ethics Committee of Ahwaz University of Medical Science, by ID number of AJUMS.REC.1393.119.

### Data Collection

We assessed the health records of pregnant women who gave birth in summer, 2015 and interview them to gather medical, health care, social and demographic data.

We used the data of national program of mother’s health care and also health records to gather data. In national program of mother’s health care, CBC test including hemoglobin (Hb) and hematocrit (Hct) were taken for all pregnant women, in first (6–10^th^ wk) and third (26–30^th^ wk) trimester. The pregnant women with Hb<11 g/dl are anemic and Hb≥11 g/dl are normal in both first and third trimester of pregnancy. Furthermore, based on serum Hb concentration, anemia categorized in three levels; mild (10–10.9 g/dl), moderate (7–9.9 g/dl), and severe (<7 g/dl).

Moreover, we used a structured questionnaire to gather medical, health care, social and demographic data including; geographical class of MMR (low, moderate, or high), resident area, age, women educational level, husband’s educational level, women occupation, husband’s occupation, number of previous pregnancies, number of children, pre-pregnancy care, the adequacy of care, terms of pregnancy (wanted or un-wanted), body mass index, total risk and diseases in current and previous pregnancies, abortion history, folate supplementation, iron supplementation, type of house, home ownership, and private car.

Total risk and diseases in current and previous pregnancies which asked in questionnaire were including preeclampsia/eclampsia, placental abruption, Placenta previa, multiple pregnancies, bleeding after delivery, late delivery, pre-term delivery, hard delivery, fast delivery, recurrent miscarriage, caesarean, stillbirth, ectopic pregnancy, neonatal death, low birth weight or macrosomia, nutritional deficiencies, congenital disorders, chronic disease, and drug allergies.

The validity of the questionnaire was confirmed by seven experts in this field. The reliability of the questionnaire was also assessed and the Cronbach’s alpha coefficient was estimated at 0.89.

Statistical analyses were done using the SPSS software package, version 16.0 (SPSS Inc., Chicago, IL, USA). Chi-square test was employed to determine the differences in variables between the normal and anemic groups. Moreover, backward stepwise logistic regression analyses were used to relate the underlying factors to the anemia status. The numerical data are expressed as mean ± SD and categorical as N (%). The statistically significant level was considered as *P*<0.05 in all statistical tests.

## Results

The data were obtained from 2737 mothers visited at least once in health centers and delivered in 2015. Of them, 2484 (90.8%) women had records of Hb and Hct in both first and third trimester. The mean Hb and Hct concentration in total population were respectively 12.70±1.37 g/dl and 38.15±3.46% in first trimester, and 11.66±1.23 g/dl and 35.57±3.24% in third trimester. Moreover, the rate of anemia in first trimester was 8.2%, of which 6.0% and 2.2% were respectively mild and moderate. Besides, 26.7% of women were anemic in third trimester, including 20.0% and 6.7% mild and moderate anemia, respectively. Moreover, severe anemia was not seen in participants.

Table 1 shows the medical, health care, social and demographic characteristics of the study subjects and the association of anemia with underlying factors in first and third trimester of pregnancy.

**Table 1:** Underlying factors of anemia in first and third trimester of pregnancy

**Variable**		**First trimester** **Hemoglobin level**					**Third trimester** **Hemoglobin level**				
		***<11 g/dl***	***≥ 11g/dl***	***Chi-square (X2)***	***df***	P-value^*^	***<11 g/dl***	***≥ 11g/dl***	***Chi-square(X2)***	***df***	P-value^*^
Geographical classes of MMR	Low	23(3.0)§	744(97.0)	117.8	2	<0.001	76(9.9)	691(90.1)	268.9	2	<0.001
	Moderate	35(4.3)	772(95.7)				181(22.4)	626(77.6)			
	High	146(16.0)	764(84.0)				407(44.7)	503(55.3)			
Resident area	Urban	108(8.5)	1160(91.5)	0.32	1	0.60	340(26.8)	928(73.2)	0.009	1	0.92
	Rural	96(7.9)	1120(92.1)				324(26.6)	892(73.4)			
Age(yr)	< 20 yr of age	10(6.1)	154(93.9)	1.07	2	0.58	53(32.3)	111(67.7)	4.51	2	0.10
	20–30 yr of age	129(8.3)	1431(91.7)				397(25.4)	1163(74.6)			
	> 30 yr of age	63(8.5)	677(91.5)				207(28.0)	533(72.0)			
Educational level	Elementary	45(8.1)	511(91.9)	0.236	3	0.97	168(30.2)	388(69.8)	7.03	3	0.07
	Guidance	43(8.1)	488(91.9)				127(23.9)	404(76.1)			
	Diploma	79(8.0)	904(92.0)				253(25.7)	730(74.3)			
	Academic education	31(8.8)	320(91.2)				102(29.1)	249(70.9)			
Husband’s education	Elementary	46(9.4)	443(90.6)	2. 29	3	0.51	144(29.4)	345(70.6)	4.32	3	0.22
	Guidance	56(7.5)	688(92.5)				198(26.6)	546(73.4)			
	Diploma	68(7.6)	823(92.4)				220(24.7)	671(75.3)			
	Academic education	28(9.3)	272(90.7)				86(28.7)	214(71.3)			
Occupation	Housewife	182(8.0)	2091(92.0)	0.145	1	0.65	595(26.2)	1678(73.8)	1.77	1	0.19
	Occupied	14(8.9)	144(91.1)				49(31.0)	109(69.0)			
Husband’s occupation	Occupied	173(8.3)	1914(91.7)	0.450	1	0.58	549(26.3)	1538(73.7)	1.43	1	0.23
	Jobless	24(7.2)	309(92.8)				98(29.4)	235(70.6)			
Number of previous pregnancies	None	54(6.5)	778(93.5)	9.45	3	0.02	205(24.6)	627(75.4)	13.20	3	0.004
	Once	83(9.8)	762(90.2)				217(25.7)	628(74.3)			
	Twice	34(6.9)	460(93.1)				132(26.7)	362(73.3)			
	Three times and	32(10.5)	273(89.5)				107(35.1)	198(64.9)			
	more										
Number of children	No children	59(6.3)	883(93.7)	11.92	3	0.008	225(23.9)	717(76.1)	23.67	3	<0.001
	1	83(8.9)	854(91.1)				245(26.1)	692(73.9)			
	2	35(8.4)	380(91.6)				115(27.7)	300(72.3)			
	3+	19(14.3)	114(85.7)				58(43.6)	75(56.4)			
Pre-pregnancy care	No Care	125(11.3)	994(88.8)	25.68	2	<0.001	334(30.7)	776(69.3)	21.01	2	<0.001
	Once	70(6.0)	1102(94.0)				287(24.5)	885(75.5)			
	Twice and more	8(4.2)	184(95.8)				33(17.2)	159(82.8)			
The adequacy of care	Inadequate (< 6 times per pregnancy)	81(12.9)	545(87.1)	24.81	1	<0.001	206(32.9)	420(67.1)	16.30	1	<0.001
	Adequate (≥ 6 times per pregnancy)	123(6.6)	1735(93.4)				458(24.7)	1400(75.3)			
Terms of pregnancy	Did not want	35(11.1)	279(88.9)	4.41	1	0.04	90(28.7)	224(71.3)	0.957	1	0.33
	Wanted	162(7.7)	1949(92.3)				550(26.1)	1561(73.9)			
Body Mass Index	Underweight	17(12.6)	118(87.4)	6.07	3	0.10	43(31.9)	92(68.1)	7.85	3	0.04
	Normal	93(8.7)	974(91.3)				299(28.0)	768(72.0)			
	Overweight	49(7.1)	639(92.9)				159(23.1)	529(76.9)			
	Obese	19(6.5)	274(93.5)				72(24.6)	221(75.4)			
Total risk and diseases in current and previous pregnancies	No risk or disease	142(8.5)	1538(91.5)	0.396	1	0.58	462(27.5)	1218(72.5)	1.57	1	0.22
	At least one risk or disease	62(7.7)	742(92.3)				202(25.1)	602(74.9)			
Abortion history	No	170(8.5)	1832(91.5)	1.07	1	0.35	530(26.5)	1472 (73.5)	0.349	1	0.56
	Yes	34(7.1)	448(92.9)				134(27.8)	348(72.2)			
Folate Supplementation during pregnancy	1–5 times	142(7.6)	1717(92.4)	0.338	1	0.55	494(26.6)	1365(73.4)	0.984	1	0.33
	6–10 times	37(8.5)	400(91.5)				106(24.3)	331(75.7)			
Iron Supplementation during pregnancy	1–5 times	134(9.3)	1301(90.7)	6.27	1	0.01	400(27.9)	1035(72.1)	2.51	1	0.11
	6–10 times	66(6.5)	946(93.5)				253(25.0)	759(75.0)			
Type of house	Apartment	34(5.9)	542(94.1)	4.83	1	0.02	124(21.5)	452(78.5)	9.43	1	0.002
	Non-apartment	159(8.8)	1655(91.2)				508(28.0)	1306(72.0)			
Home owner-ship	Personal	137(8.7)	1430(91.3)	2.35	1	0.13	418(26.7)	1149(73.3)	0.152	1	0.73
	Impersonal	59(7.0)	789(93.0)				220(25.9)	628(74.1)			
Private car	Yes	87(7.8)	1033(92.2)	0.127	1	0.76	293(26.2)	827(73.8)	0.042	1	0.85
	No	104(8.2)	1170(91.8)				338(26.5)	936(73.5)			

§ Data are presented as number (%),

* *P*-value resulted from Pearson chi-square Test

In addition, Table 2 reports the determinants of anemia in pregnant women achieved from regression analysis. The strongest protective factors in first trimester were geographical classes with low and moderate MMR, lower number of previous pregnancies, pre-pregnancy care once time and more, and adequate care, also high number of children more than two and non-apartment type of house were identified as risk factor. Moreover, in third trimester of pregnancy, geographical classes with low and moderate MMR, pre-pregnancy care once time and more, and higher body mass index were protective factors, and non-apartment type of house was risk factor of anemia.

**Table 2:** Backward stepwise logistic regression analyses to relate medical, health care, social, and demographic factors with anemia in first and third trimester of pregnancy

***Variable***	***Third trimester***			***First trimester***		
	***OR***	***95% CI***	***P-value***	***OR***	***95% CI***	***P-value***
Geographical classes of MMR			< 0.001			< 0.001
High	2.564	1.865–3.694		2.531	1.901–3.527	
Low and moderate (Ref.)	1			1		
Number of previous pregnancies					^*^	
0–1 (Ref.)	1					
1+	2.077	1.666–3.700	0.01			
Number of children			0.01			0.06
0–2 (Ref.)	1			1		
2+	2.07	1.13–3.80		1.26	0.98–1.61	
Pre-pregnancy care			0.001			0.003
No Care	1.717	1.249–2.362		1.367	1.109–1.684	
Once and more (Ref.)	1			1		
The adequacy of care			0.01		^*^	
Adequate (Ref.)	1					
Inadequate	1.501	1.080–2.086				
Type of house			0.02			0.02
Apartment (Ref.)	1			1		
Non-apartment	1.57	1.04–2.36		1.33	1.03–1.72	
Body Mass Index		^*^				0.01
Underweight and normal				1.288	1.043–1.591	
Overweight and obese (Ref.)				1		

Ref; Reference category,

* Variable not determined in logistic regression models

## Discussion

The present study revealed 8.2% and 26.7% of women were anemic in first and third trimester of pregnancy, respectively. Hence, The associated factors of anemia in pregnant women were identified as geographical classes with high MMR, high number of previous pregnancies, high number of children, no care prior to pregnancy, inadequate care, non-apartment type of house, and lower body mass index.

This study revealed anemia (Hb<11 g/dl) in 8.2% of women in first trimester and higher than one-quarter of women in third trimester of pregnancy. Similarly, 16.8% of pregnant women were anemic in Iran (11). We found lower frequency of anemia in Iranian pregnant women compared to some other developing countries. For instance, this rate was reported 37% in India (12), 40% in Bangladesh (13), 42.5% in Pakistan (14), 42.7% in south of Africa (15), and 70% in China (16). The lower frequency of anemia in Iranian pregnant women may be due to socioeconomic development and nutritional improvement. Furthermore, the high coverage of primary health care in all regions of Iran, especially in rural areas, facilitated access to free services which effectively ameliorated the anemia.

This study demonstrated an increasing trend of anemia between first and third trimester of pregnancy. Chowdhury et al. were also reported the same results (12). Anemia exacerbated during pregnancy as a result of increased blood volume and gradual reduction of body iron storage. Moreover, pica and anorexia in some women may contribute to the anemia through lower in-take of foods (3, 17). We found significant variation in prevalence of anemia between provinces with different MMR. As the rate of anemia in high risk provinces were higher than other regions nearly more than four times in first and more than three times in third trimester of pregnancy.

In this regard, recent literature reported different prevalence of anemia in various region of a country. A systematic review showed higher rate of anemia in center of Iran (23%) and lower level (12%) in west region (18). This study was also reported lower odds of anemia in geographical classes with low MMR which placed near west region of Iran. In addition, this rates as 12.1% in Hoima to 32.9% in Gulu from Uganda (19). These results may be due to differences in socioeconomic status, culture and ethnicity, lifestyle, health service utilization, etc. However, lower family income or poor economic status and low social level are the most determinants of high prevalence of anemia (19). Although we did not assess the family income, we use two variables which indirectly demonstrate economic status; personal car and home ownership. Anyway, the association of these factors with anemia were not statistically significant.

This study demonstrated pre-pregnancy care could effectively prevent anemia during pregnancy. According to health ministry protocol (20) in primary health care programs, all women in childbearing age should take health care at least once a year to control weight, blood pressure, etc. Therefore, they gained necessary training to prepare for a low risk or safe pregnancy. Consequently, the positive role of pre-pregnancy care in control of anemia can be achieved by both health care services and self-care of people. Dietary inadequacy of iron and inappropriate use of supplements was reported as underlying cause of anemia in pregnant women (21).

Iron supplementation in concomitant with folic acid is a preventive strategy to control anemia during pregnancy and after childbirth (22–24). This study population started intake of folic acid and iron supplements respectively from early pregnancy and 16^th^ week (20). However, folic acid and iron supplementation could not prevent anemia. It may be due to irregular intake or consumption of foods impaired absorption of iron like tea or nutrients such as calcium.

## Conclusion

The frequency of anemia in pregnant women differ in various parts of Iran and is at a higher level in provinces with a high MMR. The frequency of anemia in third trimester was more than double of the first trimester of pregnancy. Some determinants of anemia were including geographical classes with high MMR, higher number of previous pregnancies, higher number of children, no care prior to pregnancy, inadequate care, non-apartment type of house, and lower body mass index. Given the increasing trend of prevalence of anemia from 2005 (18), we propose more and updated educational training for health staff to achieve an effective performance in health care programs. Moreover, more researches is needed on the quality and quantity of folic acid and iron supplementation and their effectiveness.

## Ethical considerations

Ethical issues (Including plagiarism, informed consent, misconduct, data fabrication and/or falsification, double publication and/or submission, redundancy, etc.) have been completely observed by the authors.
